# Phenolic Profiles of Leaves, Grapes and Wine of Grapevine Variety Vranac (*Vitis* *vinifera* L.) from Montenegro

**DOI:** 10.3390/foods9020138

**Published:** 2020-01-28

**Authors:** Danijela Šuković, Bojana Knežević, Uroš Gašić, Milica Sredojević, Ivanka Ćirić, Slavica Todić, Jelena Mutić, Živoslav Tešić

**Affiliations:** 1 The Centre for Ecotoxicological Research, Bulevar Sarla de Gola 2, 81000 Podgorica, Montenegro; danijela.sukovic@ceti.co.me (D.Š.); bojana.knezevic@ceti.co.me (B.K.); 2 Institute for Biological Research “Siniša Stanković”—National Institute of Republic of Serbia, University of Belgrade, Bulevar despota Stefana 142, 11060 Belgrade, Serbia; uros.gasic@ibiss.bg.ac.rs; 3 Innovation Center of the Faculty of Chemistry, University of Belgrade, P.O. Box 51, 11158 Belgrade, Serbia; pantelicm@chem.bg.ac.rs (M.S.); ivankai@chem.bg.ac.rs (I.Ć.); 4 Faculty of Agriculture, University of Belgrade, Nemanjina 6, 11080 Belgrade, Serbia; slavicat@agrif.bg.ac.rs; 5 Faculty of Chemistry, University of Belgrade, P.O. Box 51, 11158 Belgrade, Serbia; jmutic@chem.bg.ac.rs

**Keywords:** vranac, grape, wine, LC/MS, polyphenolics, anthocyanins

## Abstract

Vranac, an old autochthonous red grapevine variety of Montenegro, was first mentioned in a historical document published in the 15th century. As currently the study of indigenous varieties is of particular importance, the subject of this work was detailed characterization of phenolic compounds in the autochthonous grapevine variety Vranac, from the Montenegrin Podgorica subregion. Phenolic profiles of leaves, berries (skin, seeds, and pulp were examined separately) and young monovarietal wine were determined using ultra-high performance liquid chromatography (UHPLC) with linear trap quadrupole (LTQ)—Orbitrap XL mass spectrometry (MS). Total phenolic content (TPC) and radical scavenging activity (RSA) were higher for the grape seeds extracts, followed by extracts of grape skins and pulps. As expected, the total anthocyanin content (TAC) was higher in grape skin than in wine. A total of one hundred twenty nine compounds (forty two phenolic acids and their derivatives, twenty three flavan-3-ols, twenty one flavanols, five stilbenes and thirty eight anthocyanins) were identified in the investigated extracts. To our best knowledge, this is the first report of tentative identification of (epi)catechin 3-*O*-coumarate in grape seed and chalcan-flavan 3-ol dimers in wine and grape seed.

## 1. Introduction

Content of phenolic compounds is one of the main factors in the quality of grapes and wine. Quantity and structure of phenolic compounds in grapes significantly affect the oenological potential of grapes and sensory attributes of the wine, influencing the color, astringency, bitterness, stability and age-ability of wines [1].

Different factors affect the polyphenolic profile, such as variety [2,3], climatic conditions and seasonal weather [4,5], vine health status [6], as well as vineyard management [7]. In addition, the polyphenols profile of wine is influenced by the winemaking techniques employed [5].

Bearing in mind the importance of the phenolic grape profile on the quality and sensory properties of wines and numerous factors affecting the phenolic profile, great interest in these compounds is quite justified [8]. Phenolic biological properties such as antioxidant, anti-inflammatory, cardioprotective, and cancer protective effects in human medicine have been studied [9]. The chemical nature and behavior of polyphenols contribute to the fingerprint of authenticity and typicality of the variety and area of origin [10].

The anthocyanin fingerprints of varietal wine have been suggested as an analytical tool for authenticity certification [11]. On the other hand, because the anthocyanin profile of the wine may be different from the anthocyanin profile of the grape and can be influenced by the wine production techniques applied, some authors challenge the use anthocyanin pattern of the grape skin and corresponding wine for this purpose [12]. Dimitrovska et al. [11] state that anthocyanin profile of the grape berry skin and produced wine is similar and the correlation between the grape and wine anthocyanin patterns could be confirmed. Some other factors such as the age of the wine may compromise the use anthocyanin fingerprinting in assessment of authenticity of wine.

The indigenous Balkan wine grape variety Vranac is widely spread in Montenegro, Serbia and mostly in North Macedonia where it represents a very important variety from an economic point of view [3].

The aim of this study was detailed characterization of the phenolic profile of the autochthonous grapevine variety Vranac, originating from the Podgorica subregion (Montenegro) using ultra-high performance liquid chromatography (UHPLC) in combination with high-resolution and multi stage mass spectrometry (MS). The composition of polyphenols was determined in leaves, different parts of berry (seed, skin, pulp), as well as in the young monovarietal wine with a view to promoting the varietal character of Vranac young wine and to advising on the most appropriate winemaking techniques. Further, detailed analysis of the phenolic profile of Vranac variety, with the already existing ampelographic and molecular characterization will contribute to a more complete characterization of this autochthonous variety.

## 2. Materials and Methods

### 2.1. Chemicals

Acetonitrile, methanol and formic acid (all MS grade), standards of phenolic compounds, and 6-hydroxy-2,5,7,8-tetramethylchroman-2-carboxylic acid (Trolox) were obtained from Sigma-Aldrich (Steinheim, Germany), while 2, 2-diphenyl-1-picrylhydrazyl (DPPH˙) was purchased from Fluka AG (Buch, Switzerland). Folin-Ciocalteu reagent and sodium carbonate were obtained from Merck (Darmstadt, Germany). Standard solutions and dilutions were prepared using ultrapure water (TKA Germany MicroPure water purification system, 0.055 µS/cm). Syringe filters (25 mm, nylon membrane 0.45 μm) were purchased from Supelco (Bellefonte, PA, USA).

### 2.2. Samples

The Vranac berry samples were collected at technological maturity (September 2014) from three vineyards located at the “Ćemovsko” field location which belongs to the Montenegrin viticultural region. Leaf samples were collected from the same places as the grape samples from which the tested wine was made. The Geographic coordinate system (GPS) coordinates of the experimental site is 42.26° N, 19.16° E and elevation 40–70 m a.s.l. A sketch of all three vineyards is shown on Figure S1. The proximity of the sea to a large extent determines the mesoclimate of the “Ćemovsko” field. Sampling at “Aerodrom” location was performed in block IV at T2, T3 and T4. Sampling at the location “Šipčanik” was realized on T10, T11 and T12. Sampling at “Bunar” location was realized in Block II, T1. For the sample abbreviations see Table 1. This region is located on the southernmost part of the Adriatic coast. Sampling was performed by picking grape berries randomly distributed throughout different vines. Each sample consisted of three replicates, each of 100 randomly selected berries on both sides of canopy and from different parts of clusters. Before the analysis, frozen grape berries were separated into three tissues (skin, pulp, and seeds). The seeds, as well as leaf samples were washed with water and dried (in the dark, at 22 °C) for twenty days. Dry samples were ground into a powder.

### 2.3. Preparation of Extracts and Fractionation of the Skin Extract and Wine

Procedures for extraction of phenolic compounds from grapes and leaves were described in literature [13,14]. Dry leaf and seed samples were extracted with MeOH/H_2_O (70:30, *v*/*v*) solution containing 0.1% HCl, while frozen grape skins and pulps were extracted with acidified MeOH (0.1% HCl *v*/*v*). Extractions (in an ultrasonic bath during 60 min, at room temperature, in shade) were repeated three times. All three fractions were merged into one total extract and evaporated (at 40 °C) on a vacuum evaporator IKA RV 8 (IKA^®^-Werke GmbH and Co. KG, Staufen, Germany) to dryness. The residues were dissolved in MeOH/H_2_O (60:40, *v*/*v*) solution, filtered, and analyzed using UHPLC—LTQ Orbitrap MS.

Solid-phase extraction (SPE) was used in order to separate anthocyanins from wine sample and grape skin extracts. C18 Sep-Pak cartridges (Phenomenex, Torrance, CA, USA) were preconditioned with 3 mL of acidified (0.1% HCl) methanol (HPLC grade) and 9 mL of ultrapure water. A total of 2 mL of sample (previously prepared skin extract and wine) was passed through cartridge, and then the cartridge was washed with 6 mL of ultrapure water to remove all remain sugars and other polar constituents. The adsorbed anthocyanins were eluted with acidified methanol (1 mL). Prior to UHPLC—LTQ Orbitrap MS analysis, the prepared extracts were filtered through a 0.45 µm nylon membrane filter.

### 2.4. Spectrophotometric Determination of Total Phenolic Content (TPC), Radical Scavenging Activity (RSA), and Total Anthocyanin Content (TAC)

A Cintra 6 UV-VIS spectrometer (GBC Scientific Equipment Ltd., Hampshire, IL, USA) was used in all spectrophotometric measurements. TPC was determined using Folin-Ciocalteu reagent, while RSA was measured using DPPH˙ reagent (procedures were described in Pantelić et al. [13]). Gallic acid was used as a standard for total phenolic determination, while the calibration curve for antioxidant measurements was obtained using Trolox. TPC values were expressed as g gallic acid equivalent (GAE) per kg of frozen (skins and pulps) and dried weight (seeds and leaves), as well as g GAE per L of wine. The results for RSA were expressed as mmol of Trolox equivalents (TE) per kg of frozen (skins and pulps) and dried (seeds and leaves) sample, or as mmol TE per L of wine. The pH-differential method, described by Pantelić et al. [15], was used in order to define TAC in grape skins and wine. For the purposes of this measurements, buffers of pH 1.0 (KCl, 0.025 mol/L) and pH 4.5 (NaOAc/HOAc, 0.4 mol/L) were used. The results obtained for TAC were expressed as g malvidin-3-glucoside (mal-3-glu) equivalents per kg of frozen grape skin weight and per L of wine, respectively. All measurements were performed in triplicate, and expressed as mean values ± standard deviation (SD).

### 2.5. UHPLC Orbitrap MS Analysis

All experiments were performed using a Thermo Scientific ultra-high performance liquid chromatography (UHPLC) system consisting of a quaternary Accela 600 pump and Accela Autosampler, connected to a linear ion trap-orbitrap (LTQ Orbitrap XL) hybrid mass spectrometer with heated-electrospray ionization probe (HESI-II, ThermoFisher Scientific, Bremen, Germany).

Separations were performed on a Syncronis C18-column (100 × 2.1 mm, 1.7 µm particle size) from Thermo Fisher Scientific. The same composition of the mobile phase and the gradient program were used for identification of non-anthocyanins in negative ionization mode and anthocyanins in positive ionization mode. The mobile phase consisted of (A) water + 0.1% formic acid and (B) acetonitrile + 0.1% formic acid. A linear gradient program was as follows: 5% B in the first 1.0 min, 1.0–14.0 min 5–95% B, 14.0–14.2 min from 95% to 5% B, and 5% B until the 20 min. The injection volume was 5 µL and flow rate was 0.3 mL min^–1^. Settings of dynamic exclusion and other ion source parameters were as previously described by Pešić et al. [16]. The normalized collision energy of the collision induced dissociation (CID) cell was set at 35 eV. The MS spectra were acquired by full range acquisition covering the *m/z* range 100–1500.

Compounds were identified in the samples according to their mass spectra, exact mass, characteristic fragmentation, and characteristic retention time. Xcalibur software (version 2.1, Thermo Fisher Scientific, Waltham, MA, USA) was used for instrument control, data acquisition and data analysis. The molecule editor program, ChemDraw (version 12.0, CambridgeSoft, Cambridge, MA, USA), was used as a reference library to calculate the exact (monoisotopic) masses of compounds of interest. The data-dependent MS^2^ events were always performed on the most intense ions detected in the full scan MS. Full scan analysis was employed to detect the monoisotopic mass of unknown compounds, while the fragmentation pathway was obtained by MS^4^.

## 3. Results and Discussion

### 3.1. TPC, RSA, and TAC

According to the results obtained for TPC in grape berries (Table 1), it can be seen that these phytochemicals were most abundant in grape seeds (51.72–81.07 g GAE/kg), followed by grape skins (7.95–14.01 g GAE/kg), while the lowest concentrations of polyphenols were found in the pulps (0.28–0.82 g GAE/kg). Obtained results are in accordance with those published by Pantelić et al. [13], where polyphenolic profiles of seven red grapevine varieties from Serbia were analyzed. The same trends were also obtained for RSA values. The highest antioxidant activity was found in grape seeds (478.05–740.25 mmol TE/kg), followed by the grape skins (52.15–88.19 mmol TE/kg) and pulps (11.48–12.30 mmol TE/kg). RSA and TPC values were compared using correlation analysis (MS Excel, Microsoft Office 2007 Professional) and a statistically strong linear relationship was found between them (*r* = 0.99).

Regarding the wine sample, TPC and antioxidant activity were 2.36 g GAE/L and 12.61 mmol TE/L, respectively. The result obtained for TPC in wine obtained herein was in agreement with those reported for Montenegrin ‘Vranac’ in a previous publication [17], but somewhat higher compared to the result reported by Radovanović et al. [18] and Matić et al. [19], and lower than those published by Radovanović et al. [20] and Mitić et al. [21]. Mitić et al. [21] examined ‘Vranac’ wines with different geographical origins, and the values obtained for RSA ranging from 13.00 to 15.02 mmol TE/L. The result of the current study was within this range.

As for the grape leaves (Table 1), TPC was in the range from 28.98 to 44.01 g GAE/kg, while RSA values ranged between 141.77 and 241.39 mmol TE/kg. Values for TPC in leaves sample are in agreement with the one reported in a previous publication [14].

TAC was determined in grape skins and wine. The obtained results indicated that TAC varied little among the examined skin samples (the average value was 5.73 g mal 3-glu/kg). In wine sample, TAC was 0.13 g mal 3-glu/L, which is in accordance with data presented by Pantelić et al. [22].

### 3.2. Polyphenolic Profiles of Vranac Leaves, Grapes and Wine

For the detailed LC/MS characterization of phenolic phytochemicals in the Vranac grapevine variety, one grape sample (seed, pulp, and skin), one leaf sample, and a sample of Vranac wine were selected (sample T1B4 Aerodrom). UHPLC-Orbitrap MS^4^ analysis of Vranac leaf, grape, and wine resulted in the detection of 91 compounds in negative ionization mode (Table 2) and 38 in positive ionization mode (Table 3).

The identified compounds represented five structurally distinct groups: (I) phenolic acids and their derivatives (42 compounds); (II) flavan-3-ols (23 compounds); (III) flavanols (21 compounds); (IV) stilbenes (5 compounds), and (V) anthocyanins (38 compounds). Among all identified compounds, twenty nine were confirmed using standards, while the others were identified by exact mass search of their deprotonated molecule [M−H]^−^, MS^2^, MS^3^, and MS^4^ fragmentation behavior, as well as by comparison with the available literature [16,23,24]. The peak numbers, retention times (*t*_R_, min), compound names, molecular formulas, calculated and exact masses ([M−H]^−^/M^+^, *m/z*), mean mass accuracy errors (ppm), major MS^2^, MS^3^, and MS^4^ fragment ions (*m/z*), as well as presence of selected compound in investigated extracts are summarized in Table 2 and Table 3. Anthocyanins were analyzed only in the Vranac wine and grape berry skin extract.

#### 3.2.1. Phenolic Acids and Their Derivatives

Phenolic acids (hydroxycinnamic and hydroxybenzoic acid derivatives), commonly present in grape and wines [25], were found as free and in the form of glycosides (pentoside and hexoside derivatives) and esters with tartaric, treonic, shikimic and quinic acids. Specifically, 32 phenolic acids and their derivatives were found in analyzed wine and leaf samples, while in seed, skin, and pulp a total of 20, 11, and 9 (respectively) compounds were identified. Protocatechuic acid (compound **3**), caffeoyltartaric (caftaric) acid (compound **4**), hydroxybenzoic acid hexoside (compound **6**), coumaryltartaric (coutaric) acid (compound **11**) and vanillic acid (compound **28**) were identified in all tested samples (Table 2). Compounds **7**, **9**, **14** and **30** were identified only in leaf samples, while compounds **12**, **18**, **22** and **24** were identified in wine samples only. Chlorogenic acid, as a hydroxycinnamic acid that was detected in leaves sample only, in previous studies has been associated with the reduction of oxidative stress conditions [26].

Presence and the content of ellagic acid (**35**) in various parts of the plant issue have attracted the attention of researchers. Ellagic acid is considered as a compound that exhibits a wide range of biological activities and could be beneficial for human health. In this research, ellagic acid pentoside (**22**) was detected only in leaves extracts, which supports the claims that the grape leaf extract has high nutritional value and can be used as supplements in human diet [14].

Two isomeric coumaryltartaric acids, **11** and **17**, with identical molecular ion ([M−H]^−^ at 295 *m/z*), but showing slightly different MS fragmentation patterns, were identified at 4.95 and 5.36 min, respectively. Compound **11** generated MS^2^ base peak at 163 *m/z* (mass of deprotonated coumaric acid), while compound **17** gave MS^2^ base peak at 149 *m/z* (mass of deprotonated tartaric acid). In addition, feruoyltartaric (fertaric) acid (compound **21**) and caffeoyltreonic acid (compound **26**) were identified in wine and leaf samples. Compound **29** at 5.99 min and 335 *m/z* giving MS^2^ base peak at 179 *m/z* (corresponding to deprotonated caffeic acid) and MS^2^ secondary peak at 135 *m/z* was identified as caffeoylshikimic acid. Compounds **1** and **18**, with same accurate masses (315 *m/z*) and similar fragmentation patterns, were marked as dihydroxybenzoic acid hexoside isomers. The first isomer at 2.89 min was identified only in grape berry (skin, pulp, and skin), while the second was identified only in wine.

#### 3.2.2. Flavan-3-ols

From the group flavan-3-ols, all identified compound (monomers, dimers, trimers) were found in grape seed samples and their identification was largely based on the evaluated MS fragments and previously reported data about phytochemicals found in wine and grapes [27]. Presence of compounds **47** (gallocatechin), **54** (catechin), **56** (epigallocatechin), **60** (epicatechin), and **62** (catechin 3-*O*-gallate) were confirmed using available standards.

In the seeds, as the richest source of flavan-3-ol, twenty three compounds were identified. The skin is known to be poorer in these compounds, and only four compounds have been identified (**48**, **54**, **56**, and **60**). Of all the identified flavan-3-ol from the seed, seventeen flavan-3-ols were also identified in the wine sample. A match in flavan-3-ols between samples of seed and wine was also expected, given the extraction of phenolic compounds from the seed into the wines during the maceration process. Unlike flavan-3-ols from the seed, all flavan-3-ols detected in the skin samples (total of four compounds) were also identified in the wine.

The results of the examination of phenolic composition of leaves of Vranac grapevine variety and another nine red wine varieties from different Serbian wine-growing regions indicate the variation in the profile of flavan-3-ols in leaves of different varieties [14].

**Figure 1:**
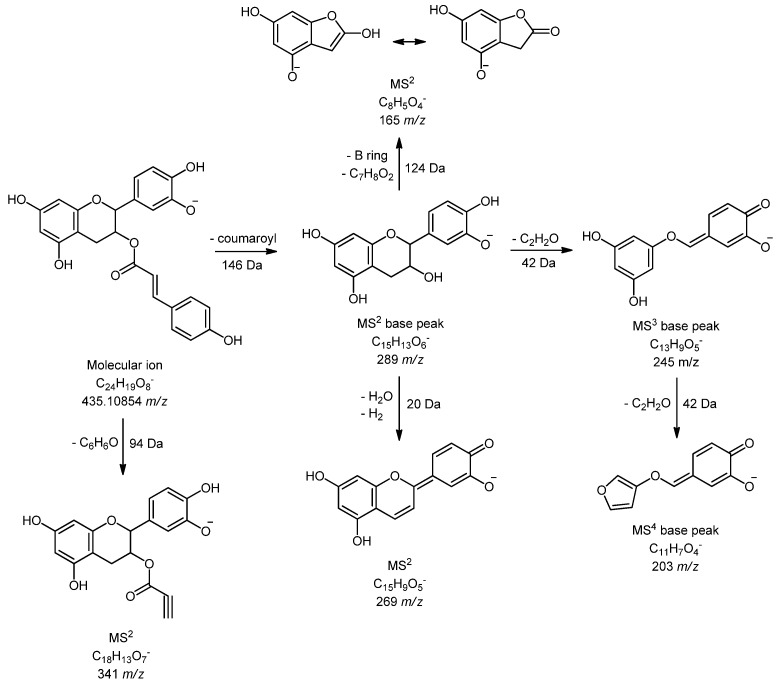
Fragmentation pathway of (Epi)catechin 3-*O*-coumarate (**65**) in negative ionization mode.

Compounds **43** (4.14 min and 451 *m/z*) and **46** (4.81 min and 451 *m/z*) with the same fragmentation pathway were identified only in grape seed, as (epi)catechin hexoside isomers. Both compounds generated MS^2^ base peak at 289 *m/z*, corresponding to deprotonated catechin or epicatechin. It is interesting to note that some compounds that are not common for *Vitis* species, such as chalcan-flavan dimers, were found in wine and grape seed samples. Two isomers of chalcan-flavan-3-ol (compounds **51** and **58**, molecular ion at 579 *m/z*) with same fragmentation pathway were identified at 5.07 and 5.59 min, respectively, and their fragmentation was in accordance with literature data [28]. Compound **65** at 8.12 min and molecular ion 435 *m/z* was marked as (epi)catechin 3-*O*-coumarate. It produced MS^2^ base peak at 289 *m/z* and further fragmentation confirmed that this was an ester of catechin and coumaric acid (Figure 1). These compounds were already isolated and identified in green tea extracts [29], and as far as we know, they have not been identified in some *Vitis* species until now. In this study, it was found only in grape seeds extract of the autochthonous grapevine variety Vranac. A very similar fragmentation pattern was noticed for (epi)catechin 3-*O*-vanillate (**64**) and it was recently identified in grape seed and red wine by Ma et al. [30].

#### 3.2.3. Flavanols

Among twenty one flavanols, six were identified using available standards (myricetin—**67**, quercetin 3-*O*-(6”rhamnosyl)glucoside—**70**, quercetin 3-*O*-galactoside—**73**, kaempferol 3-*O*-glucoside—**76**, quercetin—**85**, and kaempferol—**86**). Compound **73** (quercetin 3-*O*-galactoside) and compound **79** (quercetin 3-*O*-hexuronide) were identified in all investigated samples. It is well known that various derivatives of flavanol aglycones and glycosides are commonly present in grapes and wines [25]. For example, syringetin 3-*O*-hexoside (compound **77**), eluted at 6.99 min with molecular ion at 507 *m/z*, was confirmed by examination of its MS data. It is well known that this compound is specific to *Vitis* species because it was previously identified in grape samples [31]. Compound **81** (quercetin 3-*O*-(6”-malonyl)hexoside), found only in Vranac leaf sample at 7.36 min and 549 *m/z*, gave MS^2^ base peak at 505 *m/z* (loss of CO_2_—44 Da) and MS^3^ and MS^4^ spectrum which corresponded to the fragmentation of quercetin.

A large number of compounds from the flavanol group were present in the skin sample. Of the 21 identified flavanols, 14 were present in the skin while 4 compounds were present in the seeds (**73**, **79**, **78**, and **85**).

According to previous research, quercetin and kaempferol derivatives are the main leaf flavonoids found in *Vitis vinifera* species [32] and our research also supports this knowledge. Of the total number of detected flavanols (a total of 21 compounds), only two (**77** and **78**) were not detected in the leaves, and five compounds (**74**, **75**, **81**, **83**, and **84**) were present only in leaves. None of the flavanol derivatives that were detected solely in the leaves were detected in the grape skin sample. It is believed that the grape leaves are rich in polyphenols, specifically flavonoids, since these compounds exhibit UV protective effects and can protect chloroplast from the damaging effects of UV rays [33].

The presence of kaempferol 3-*O*-hexuronide methyl ether and kaempferol 7-*O*-(6-malonyl)hexoside (**83** and **84**) in the leaves samples of Vranac grown in Montenegro (Mediterranean climate) is contrary to the results from the Central Serbia region, were kaempferol compounds were not identified in Vranac leaves [14]. These discrepancies may be due to different climatic conditions between growing regions that influence the synthesis of these compounds.

#### 3.2.4. Stilbenes

As for stilbene derivatives, aside from trans-resveratrol, confirmed using available standard, four other derivatives were identified using exact mass search and MS fragmentation. Compound **87** with retention time 7.11 min and 389 *m/z* was identified as resveratrol hexoside. It generated MS^2^ base peak at 277 *m/z*, which was obtained by the loss of one sugar unit (hexosyl residue—162 Da). MS^3^ base peak at 227 *m/z* confirmed the presence of resveratrol as aglycone. Polymeric stilbene derivatives are common for the genus *Vitis* [34] and in our samples of grape seeds and wine it was possible to identify resveratrol dimer (compound **90**, 453 *m/z*), trimer (compound **91**, 679 *m/z*) and tetramer (compound **89**, 905 *m/z*).

#### 3.2.5. Anthocyanins

Monomeric anthocyanins derived from grape skin are of the highest importance for the color of young red wines. Consequently, the composition of free anthocyanins in grapes determines the composition of the anthocyanin profile of the produced red wines to a significant extent [35].

The most abundant compounds found in the *Vitis vinifera* red wines are malvidin derivatives and the main monomeric anthocyanins are the 3-*O*-monoglucosides. The largest number of compounds found in Vranac wine and berry skin extracts are malvidin derivatives (19 compounds), followed by peonidin (7 compounds) and delphinidin (6 compounds) derivatives. It is known that among monomeric anthocyanins, malvidin 3-*O*-glucoside and its derivatives are usually the most abundant and are the main source of the red color in very young red wines [35].

The presence of diglucoside anthocyanins in non-*V. vinifera* grape and wine is common, both 3-*O*-monoglucoside and 3,5-*O*-diglucoside of anthocyanins can be present [13]. Diglucosides are more stable than their monoglucoside derivatives but are more susceptible to browning and are less colored [36]. Some previous investigations have detected the presence of 3,5-*O*-diglucosides in grape or wine of *V. vinifera* varieties [13]. We also identified peonidine 3,5-di-*O*-hexoside (**1’**) in the Vranac skin sample. It represented the major diglucoside in wine from non-*V. vinifera* grapes. The same diglucoside has not been identified in Vranac wine. In addition, malvidin 3,5-di-*O*-glucoside (**14’**) was also identified in the skin and wine sample.

In addition, several acylated anthocyanin derivatives were found in investigated extracts. For example, malvidin 3-*O*-(6”-caffeoyl)hexoside (**22’**), found at 6.39 min and 655 *m/z*, showed MS^2^ base peak at 331 *m/z* (deprotonated malvidin), corresponding to loss of caffeoylhexoside residue (324 Da). Further loss of 16 Da (CH_4_) and 28 Da (CO) gave MS^3^ and MS^4^ base peaks at 315 and 287 *m/z*, respectively.

**Figure 2:**
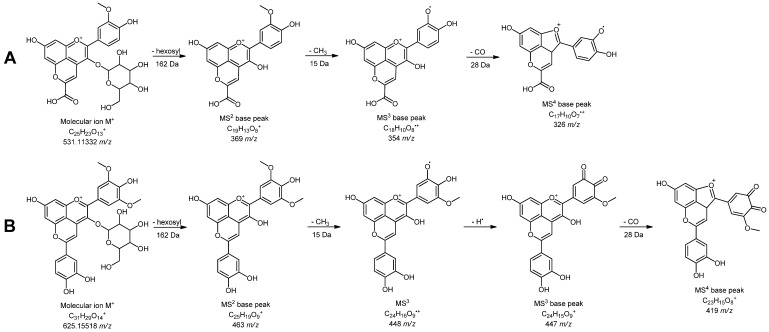
Fragmentation pathway of (**A**) peonidin 3-*O*-hexoside-pyruvate (**4’**) and (**B**) malvidin 3-*O*-hexoside-4-vinylcatechol (**24’**) in positive ionization mode.

All peonidin derivatives (except peonidin 3-*O*-hexoside-pyruvate, **4’**, Figure 2A) exhibit a specific fragment ion at 301 *m/z*, corresponding to aglycone part of molecule (Table 3).

Peonidin 3-*O*-hexoside-pyruvate was found in both investigated samples (wine and grape skin) at 5.70 min and molecular ion at 531 *m/z*. It gave a MS^2^ base peak at 369 *m/z* generated by loss of hexosyl group (162 Da). Further loss of methyl group (15 Da) gave a MS^3^ base peak at 354 *m/z*. MS^4^ base peak was formed by elimination of CO (28 Da) from A ring of anthocyanin.

In addition, a significant number of pyranoanthocyanins were found, which are known to be made by maturation and aging of wine [37]. These compounds represent stable pigments that are mostly formed during fermentation of must by reaction between free anthocyanins and certain yeast by-products (acetaldehyde, pyruvic acid and vinylphenols [38]). In our study, young wine was examined so that a large number of these compounds were not found.

Malvidin 3-*O*-hexoside-pyruvate (**18’**) or vitisin A and malvidin 3-*O*-hexoside-4-vinylphenol (**27’**) were earlier identified in Vranac wine [39]. Malvidin 3-*O*-hexoside-4-vinylcatechol or pinotin A (**24’**) was found only in wine sample at 6.76 min and 625 *m/z*. The detailed fragmentation pathway of this compound is depicted in Figure 2B.

## 4. Conclusions

In order to study in detail the polyphenolic compounds of the autochthonous grapevine variety Vranac (Podgorica region, Montenegro), the leaf, skin, seed, pulp and young Vranac wine were analyzed. To the best of our knowledge, for the first time total phenolic content and radical scavenging activity were determined in individual parts of grape berry and leaves. As expected, the highest TPC and TAC were found in seeds, followed by leaves, while the lowest values were obtained for skin and pulp.

LC-MS analyses revealed a total of 91 polyphenolic compounds (phenolic acid derivatives, flavan-3-ols, flavanols, and stilbenes) in the negative ionization mode, while in positive ionization mode 38 anthocyanins were identified. Using high-resolution mass spectrometry (HRMS) in combination with MS^n^ fragmentation, it was observed that chlorogenic acid and ellagic acid pentoside were found only in the Vranac leaf sample. The largest number of compounds from the group of flavan-3-ols (monomers, dimers, and trimers) has been identified in wine and grape seed samples. The largest number of anthocyanins, found in Vranac wine and berry skin extracts, were derivatives of malvidin.

Based on the obtained results, it can be concluded that the examined grapevine variety Vranac from Podgorica region has a wide range of diverse phenolic compounds. In addition, the results obtained in this work could give important data on the distribution of polyphenols throughout the plant (leaves and grape berries), along with wine. This information can assist in the selection of technology for the production of wines with a high amount of active ingredients.

Finally, as far as we know, tentative identification of (epi)catechin 3-*O*-coumarate in Vranac grape seed and chalcan-flavan 3-ol dimers in Vranac wine and grape seed is reported for the first time in this study. These types of compounds have not been found in any grapevine variety so far.

## Figures and Tables

**Table 1:** Total phenolic contents (TPC, g GAE/kg) and radical scavenging activities (RSA, mmol TE/kg) in the skins, seeds, pulps, and leaves of grapevine samples. Total anthocyanin content (TAC, g mal 3-glu/kg) in grape skins.

Samples		T1B4 Aerodrom	T2B4 Aerodrom	T3B4 Aerodrom	T4B4 Aerodrom	T1B2 Bunar 12	T2B2 Bunar 12	T10 Šipčanik	T11 Šipčanik	T12 Šipčanik
Skin (S)	**TPC**	8.76 ± 0.12	7.95 ± 0.08	9.77 ± 0.09	11.34 ± 0.22	12.06 ± 0.08	10.80 ± 0.09	11.75 ± 0.11	14.01 ± 0.01	11.13 ± 0.30
	**RSA**	65.17 ± 3.36	61.58 ± 1.04	52.15 ± 2.60	79.83 ± 1.29	75.86 ± 2.57	77.13 ± 0.77	77.41 ± 0.26	88.19 ± 1.55	71.32 ± 1.28
	**TAC**	5.47 ± 0.01	4.43 ± 0.01	4.26 ± 0.00	6.48 ± 0.02	7.44 ± 0.01	6.02 ± 0.05	4.95 ± 0.04	7.58 ± 0.00	4.91 ± 0.01
Pulp (P)	**TPC**	0.82 ± 0.07	0.33 ± 0.01	0.28 ± 0.00	0.51 ± 0.00	0.33 ± 0.02	0.35 ± 0.03	0.36 ± 0.01	0.42 ± 0.01	0.40 ± 0.00
	**RSA**	11.66 ± 0.25	11.97 ± 0.30	11.48 ± 0.49	12.27 ± 0.03	11.70 ± 0.05	11.82 ± 0.33	12.29 ± 0.00	12.30 ± 0.03	12.23 ± 0.10
Seed (E)	**TPC**	57.54 ± 0.91	70.58 ± 0.58	70.46 ± 0.58	61.77 ± 0.08	51.72 ± 0.83	66.23 ± 2.24	72.20 ± 0.32	73.57 ± 0.66	81.07 ± 0.40
	**RSA**	613.24 ± 17.60	655.96 ± 3.27	617.84 ± 1.63	571.63 ± 27.77	478.05 ± 3.27	673.19 ± 6.40	740.25 ± 15.24	716.18 ± 12.80	634.61 ± 25.38
Leaf (L)	**TPC**	40.30 ± 0.21	44.01 ± 0.86	41.15 ± 0.33	33.78 ± 0.85	28.98 ± 0.61	29.44 ± 0.56	31.96 ± 0.13	32.46 ± 0.17	35.44 ± 1.01
	**RSA**	201.21 ± 2.65	219.74 ± 4.68	241.39 ± 11.68	170.44 ± 6.58	163.72 ± 8.77	141.77 ± 6.07	190.92 ± 7.90	200.37 ± 0.00	185.77 ± 11.25

T—table; B—block; Aerodrom, Bunar 12, and Šipčanik—vineyards located at the “Ćemovsko” field (Montenegro).

**Table 2:** High resolution MS data and negative ion MS^2^, MS^3^, and MS^4^ fragmentation of phenolic compounds identified in Vranac wine (W), grape (seed (S), pulp (P), and skin (E)) and leaf (L) extracts.

No	*t*_R_, min	Compound Name	Molecular Formula, [M–H]^–^	Calculated Mass, [M–H]^–^	Exact Mass, [M–H]^–^	Δ ppm	MS^2^ Fragments, (% Base Peak)	MS^3^ Fragments, (% Base Peak)	MS^4^ Fragments, (% Base Peak)	W	S	P	E	L
	***Phenolic Acids and Their Derivatives***													
**1**	2.89	**Dihydroxybenzoic acid hexoside isomer 1**	C_13_H_15_O_9_^–^	315.07216	315.07135	2.57	**153** (100), 152 (50), 109 (15), 108 (10)	**109** (100)	123 (25), 109 (10), 85 (10), 81 (100)	–	+	+	+	–
**2**	3.01	**Gallic acid *^a^***	C_7_H_5_O_5_^–^	169.01425	169.01369	3.31	**125** (100)	**107** (100)	–	−	+	–	–	+
**3**	3.86	**Protocatechuic acid *^a^***	C_7_H_5_O_4_^–^	153.01933	153.01906	1.76	**109** (100), 95 (75), 79 (20), 59 (10)	**81** (100), 68 (25), 65 (15)	–	+	+	+	+	+
**4**	4.20	**Caffeoyltartaric acid**	C_13_H_11_O_9_^–^	311.04031	311.04001	0.97	179 (40), 177 (15), **149** (100)	131 (50), 103 (90), **87** (100), 59 (25)	59 (100)	+	+	+	+	+
**5**	4.60	**Gallic acid hexoside**	C_13_H_15_O_10_^–^	331.06707	331.06638	2.08	**169** (100), 125 (5)	**125** (100)	110 (10), 97 (30), 81 (100), 53 (30)	+	+	+	–	+
**6**	4.62	**Hydroxybenzoic acid hexoside isomer 1**	C_13_H_15_O_8_^–^	299.07724	299.07648	2.54	**137** (100)	**93** (100)	–	+	+	+	+	+
**7**	4.72	**Chlorogenic acid hexoside**	C_22_H_27_O_14_^–^	515.14008	515.13917	1.77	353 (40), 341 (100), 335 (25), 191 (15), 179 (45)	**179** (100), 135 (10)	135 (100)	–	–	–	–	+
**8**	4.81	**Caffeic acid hexoside**	C_15_H_17_O_9_^–^	341.08781	341.08659	3.58	191 (10), **179** (100), 135 (10)	**135** (100)	135 (100), 107 (50)	+	–	–	–	+
**9**	4.84	**Dihydroxybenzoic acid hexosyl pentoside isomer 1**	C_18_H_23_O_13_^–^	447.11441	447.11353	1.97	**315** (100), 285 (10), 153 (10)	**153** (100), 123 (10)	123 (100)	–	–	–	–	+
**10**	4.90	**Chlorogenic acid *^a^***	C_16_H_17_O_9_^–^	353.08781	353.08633	4.19	**191** (100), 179 (30), 135 (10)	173 (75), **127** (100), 111 (40), 93 (60), 85 (90)	109 (30), 99 (40), 85 (100)	+	+	–	–	+
**11**	4.95	**Coumaroyltartaric acid isomer 1**	C_13_H_11_O_8_^–^	295.04594	295.04532	2.10	**163** (100), 149 (10), 119 (5)	**119** (100)	119 (100), 93 (50)	+	+	+	+	+
**12**	4.96	**Gallic acid methyl ester**	C_8_H_7_O_5_^–^	183.02990	183.02931	3.22	**168** (100), 124 (15)	**124** (100)	95 (100)	+	−	−	−	−
**13**	5.01	**Hydroxybenzoic acid hexoside isomer 2**	C_13_H_15_O_8_^–^	299.07724	299.07593	4.38	**137** (100)	**93** (100)	–	+	–	–	–	+
**14**	5.04	**Dihydroxybenzoic acid hexosyl pentoside isomer 2**	C_18_H_23_O_13_^–^	447.11441	447.11356	1.90	**315** (100), 285 (60), 153 (50), 149 (25)	**153** (100), 123 (10)	123 (100), 109 (5)	–	–	–	–	+
**15**	5.26	***p*-Hydroxybenzoic acid *^a^***	C_7_H_5_O_3_^–^	137.02442	137.02385	4.16	109 (10), **93** (100)	**93** (100)	–	+	+	+	–	+
**16**	5.32	**Coumaric acid hexoside isomer 1**	C_15_H_17_O_8_^–^	325.09289	325.09167	3.75	**163** (100), 119 (10)	**119** (100)	–	+	–	–	+	+
**17**	5.36	**Coumaroyltartaric acid isomer 2**	C_13_H_11_O_8_^–^	295.04594	295.04501	3.15	163 (30), **149** (100), 119 (5)	131 (50), 103 (90), **87** (100), 59 (30)	–	+	–	–	–	+
**18**	5.44	**Dihydroxybenzoic acid hexoside isomer 2**	C_13_H_15_O_9_^–^	315.07216	315.07123	2.95	**153** (100), 135 (10), 109 (10)	**135** (100), 109 (50)	91 (100)	+	–	–	–	–
**19**	5.47	**3-*O*-*p*-Coumaroylquinic acid**	C_16_H_17_O_8_^–^	337.09289	337.09171	3.50	191 (10), 173 (10), **163** (100), 119 (10)	**119** (100)	–	+	–	–	–	+
**20**	5.51	**Coumaric acid hexoside isomer 2**	C_15_H_17_O_8_^–^	325.09289	325.09164	3.85	289 (20), 265 (20), 187 (40), 163 (80), **145** (100)	**117** (100)	–	+	–	–	+	+
**21**	5.62	**Feruloyltartaric acid**	C_14_H_13_O_9_^–^	325.05651	325.05518	4.09	**193** (100), 113 (5)	178 (65), **149** (100), 134 (40)	134 (100)	+	–	–	–	+
**22**	5.66	**Ferulic acid pentoside**	C_15_H_17_O_8_^–^	325.09289	325.09135	4.74	**193** (100)	178 (60), **149** (100), 134 (30)	134 (100)	+	–	–	–	–
**23**	5.66	**Ferulic acid hexoside**	C_16_H_19_O_9_^–^	355.10346	355.10199	4.14	217 (60), **193** (100), 175 (40), 134 (10)	178 (20), 149 (40), **134** (100)	134 (50), 106 (100)	+	–	–	–	+
**24**	5.67	***p*-Hydroxyphenylacetic acid *^a^***	C_8_H_7_O_3_^–^	151.04007	151.03996	0.73	**136** (100), 95 (5)	108 (25), **92** (100)	108 (100)	+	–	–	–	−
**25**	5.67	**Ellagic acid hexoside**	C_20_H_15_O_13_^–^	463.05181	463.05078	2.22	302 (15), **301** (100), 300 (50), 289 (10), 273 (10)	301 (70), 284 (20), **257** (100), 229 (65), 185 (30)	229 (50), 213 (30), 185 (100)	–	+	–	–	+
**26**	5.82	**Caffeoylthreonic acid**	C_13_H_13_O_8_^–^	297.06159	297.06030	4.34	179 (15), **135** (100)	**117** (100), 89 (90), 75 (80)	89 (100)	+	–	–	–	+
**27**	5.84	**Caffeic acid *^a^***	C_9_H_7_O_4_^−^	179.03498	179.03436	3.46	**135** (100), 117 (10), 91 (20), 59 (15)	**107** (100), 59 (50)	–	+	+	–	+	+
**28**	5.89	**Vanillic acid *^a^***	C_8_H_7_O_4_^–^	167.03498	167.03433	3.89	153 (10), 152 (80), 124 (10), **123** (100), 108 (20)	**108** (100)	79 (100)	+	+	+	+	+
**29**	5.99	**Caffeoylshikimic acid**	C_16_H_15_O_8_^–^	335.07724	335.07654	2.09	**179** (100), 135 (25)	**135** (100)	107 (100)	+	+	–	–	+
**30**	6.11	**Ellagic acid pentoside**	C_19_H_13_O_12_^–^	433.04125	433.04080	1.04	**301** (100), 300 (80)	301 (95), 284 (25), **257** (100), 229 (70), 185 (40)	229 (70), 213 (30), 201 (15), 185 (100)	–	–	–	–	+
**31**	6.20	**Syringic acid *^a^***	C_9_H_9_O_5_^−^	197.04555	197.04474	4.11	**183** (100), 153 (40), 138 (10)	**167** (100), 138 (10), 123 (5)	–	+	–	+	+	+
**32**	6.25	**Coumaric acid hexoside isomer 3**	C_15_H_17_O_8_^–^	325.09289	325.09180	3.35	289 (10), **163** (100), 161 (50), 119 (60), 101 (20)	**91** (100)	–	+	–	–	–	–
**33**	6.50	***p*-Coumaric acid *^a^***	C_9_H_7_O_3_^–^	163.04007	163.03951	3.43	**119** (100)	119 (60), 101 (20), 93 (25), **91** (100), 72 (10)	–	+	+	–	+	+
**34**	6.58	**Gallic acid ethyl ester**	C_9_H_9_O_5_^–^	197.04555	197.04480	3.81	**169** (100)	**125** (100)	107 (10), 97 (40), 81 (100)	+	+	−	−	−
**35**	6.75	**Ellagic acid *^a^***	C_14_H_5_O_8_^–^	300.99899	300.99805	3.12	284 (40), 271 (60), **257** (100), 229 (85), 185 (40)	**229** (100), 213 (20), 185 (85)	201 (100), 185 (95), 157 (30), 145 (20), 129 (10)	+	+	–	–	+
**36**	6.76	**Sinapic acid *^a^***	C_11_H_11_O_5_^−^	223.06120	223.06047	3.27	**208** (100), 179 (30), 164 (20)	193 (10), **164** (100), 149 (15), 135 (5)	149 (100), 135 (35)	−	+	–	–	−
**37**	7.05	**Ferulic acid *^a^***	C_10_H_9_O_4_^–^	193.05063	193.04995	3.52	178 (70), **149** (100), 134 (50)	**134** (100)	106 (100)	+	+	–	–	+
**38**	7.67	**Caffeoylcoumaroylquinic acid**	C_25_H_23_O_11_^–^	499.12404	499.12375	0.58	361 (5), **337** (100), 163 (10)	191 (10), 173 (60), **163** (100), 119 (10)	119 (100)	–	+	–	–	–
**39**	8.01	**Dihydroxybenzoic acid ethyl ester**	C_9_H_9_O_4_^–^	181.05063	181.05011	2.87	**153** (100), 109 (10)	**109** (100)	–	+	−	−	+	−
**40**	8.02	**Caffeic acid methyl ester**	C_10_H_9_O_4_^–^	193.05063	193.05019	2.28	178 (30), **161** (100), 134 (70), 111 (10)	**133** (100)	–	+	−	−	−	+
**41**	8.96	**Cinnamic acid *^a^***	C_9_H_7_O_2_^−^	147.04515	147.04480	2.38	104 (10), **103** (100), 87 (10)	**119** (100)	–	–	+	–	–	+
**42**	9.12	***p*-Coumaric acid methyl ester**	C_10_H_9_O_3_^–^	177.05572	177.05504	3.84	177 (10), 162 (40), **145** (100), 118 (50)	**177** (100)	–	+	−	−	+	+
	***Flavan-3-ols***													
**43**	4.14	**(Epi)catechin hexoside isomer 1**	C_21_H_23_O_11_^-^	451.12404	451.12378	0.58	**289** (100), 161 (5)	**245** (100), 205 (40), 179 (15)	227 (30), 203 (100), 187 (30), 175 (10), 161 (20)	–	+	–	–	–
**44**	4.41	**Prodelphinidin dimer B type**	C_30_H_25_O_13_^–^	593.13006	593.12958	0.81	467 (15), **425** (100), 407 (30), 289 (20)	**407** (100), 381 (5), 273 (10)	389 (30), 297 (30), 285 (100), 243 (70)	+	+	–	–	–
**45**	4.70	**Procyanidin dimer A type isomer 1**	C_30_H_23_O_12_^–^	575.11950	575.11857	1.62	449 (80), 423 (70), **407** (100), 289 (15), 287 (35), 285 (25)	389 (20), 297 (30), **285** (100), 243 (30)	257 (100), 213 (10)	+	+	–	–	–
**46**	4.81	**(Epi)catechin hexoside isomer 2**	C_21_H_23_O_11_^-^	451.12404	451.12387	0.38	**289** (100), 161 (5)	**245** (100), 205 (40), 179 (15)	227 (30), 203 (100), 187 (30), 175 (10), 161 (20)	–	+	–	–	–
**47**	4.83	**Gallocatechin *^a^***	C_15_H_13_O_7_^−^	305.06668	305.06592	2.49	261 (50), 221 (70), 219 (70), **179** (100), 165 (35)	**164** (100), 151 (40), 135 (30)	120 (100), 108 (20)	+	+	–	–	–
**48**	4.85	**Procyanidin dimer B type isomer 1**	C_30_H_25_O_12_^–^	577.13515	577.13391	2.15	559 (10), 451 (30), **425** (100), 407 (50), 289 (25), 287 (10)	**407** (100), 381 (5), 273 (10)	389 (30), 297 (30), 285 (100), 243 (70)	+	+	+	+	–
**49**	4.94	**Procyanidin dimer A type isomer 2**	C_30_H_23_O_12_^–^	575.11950	575.11850	1.74	449 (70), **423** (100), 407 (60), 289 (10), 287 (20), 285 (5)	**405** (100), 313 (35), 297 (45), 285 (25), 243 (30)	387 (10), 283 (10), 243 (100)	+	+	–	–	–
**50**	5.01	**Procyanidin trimer B type isomer 1**	C_45_H_37_O_18_^-^	865.19854	865.19684	1.96	**695** (100), 577 (60), 425 (30), 407 (30), 287 (30)	**543** (100), 451 (45), 243 (60)	525 (100), 391 (40)	+	+	–	–	–
**51**	5.07	**Chalcan-flavan 3-ol dimer isomer 1**	C_30_H_27_O_12_^–^	579.15079	579.14984	1.64	**289** (100), 245 (10)	**245** (100), 205 (30), 179 (15)	227 (30), 203 (100), 187 (30), 175 (10), 161 (20)	+	+	–	–	–
**52**	5.13	**Procyanidin dimer B type isomer 2**	C_30_H_25_O_12_^-^	577.13515	577.13403	1.94	559 (10), 451 (30), **425** (100), 407 (50), 289 (25), 287 (10)	**407** (100), 381 (5), 273 (10)	389 (30), 297 (30), 285 (100), 243 (70)	+	+	+	–	–
**53**	5.25	**Procyanidin dimer A type isomer 3**	C_30_H_23_O_12_^–^	575.11950	575.11830	2.09	449 (50), **423** (100), 407 (30), 289 (5), 287 (20), 285 (10)	**405** (100), 313 (50), 297 (30), 285 (45), 243 (40)	387 (20), 361 (20), 243 (100)	+	+	–	–	–
**54**	5.26	**Catechin *^a^***	C_15_H_13_O_6_^–^	289.07176	289.07080	3.32	271 (5), **245** (100), 205 (40), 179 (15), 125 (5)	227 (30), **203** (100), 187 (25), 175 (10), 161 (20)	188 (70), 185 (20), 175 (100), 161 (40), 157 (10)	+	+	+	+	+
**55**	5.31	**Procyanidin dimer B type isomer 3**	C_30_H_25_O_12_^-^	577.13515	577.13397	2.04	425 (25), 407 (10), 329 (10), **289** (100), 287 (80)	**245** (100), 205 (30), 179 (15)	227 (30), 203 (100), 187 (30), 175 (10), 161 (20)	+	+	+	–	–
**56**	5.36	**Epigallocatechin *^a^***	C_15_H_13_O_7_^–^	305.06668	305.06552	3.80	261 (40), 247 (20), 221 (90), 219 (80), **179** (100)	**164** (100), 151 (35), 135 (30)	120 (100), 108 (25)	+	+	–	+	–
**57**	5.39	**Procyanidin trimer B type isomer 2**	C_45_H_37_O_18_^-^	865.19854	865.19592	3.03	**695** (100), 577 (80), 425 (30), 407 (40), 287 (35)	**543** (100), 451 (45), 243 (60)	525 (100), 391 (40)	+	+	–	–	–
**58**	5.59	**Chalcan-flavan 3-ol dimer isomer 2**	C_30_H_27_O_12_^–^	579.15079	579.15002	1.33	**289** (100), 245 (10)	**245** (100), 205 (30), 179 (15)	227 (30), 203 (100), 187 (30), 175 (10), 161 (20)	+	+	–	–	–
**59**	5.64	**Procyanidin trimer B type isomer 3**	C_45_H_37_O_18_^-^	865.19854	865.19818	0.41	**695** (100), 577 (70), 425 (30), 407 (40), 287 (30)	**543** (100), 451 (45), 243 (60)	525 (100), 391 (40)	+	+	–	–	–
**60**	5.91	**Epicatechin *^a^***	C_15_H_13_O_6_^–^	289.07176	289.07085	3.15	271 (5), **245** (100), 205 (40), 179 (15), 125 (5)	227 (35), **203** (100), 187 (30), 175 (15), 161 (25)	188 (60), 185 (20), 175 (100), 161 (35), 157 (15)	+	+	+	+	+
**61**	6.20	**Procyanidin trimer B type isomer 4**	C_45_H_37_O_18_^-^	865.19854	865.19672	2.10	**695** (100), 577 (80), 425 (35), 407 (35), 287 (30)	**543** (100), 451 (45), 243 (60)	525 (100), 391 (40)	+	+	–	–	–
**62**	6.50	**Catechin 3-*O*-gallate *^a^***	C_22_H_17_O_10_^−^	441.08272	441.08209	1.43	331 (10), **289** (100), 271 (10), 169 (25)	271 (5), **245** (100), 205 (40), 179 (20)	227 (20), 203 (100), 187 (20), 175 (10), 161 (20)	–	+	–	–	–
**63**	6.68	**(Epi)gallocatechin 3-*O*-gallate methyl ether**	C_23_H_19_O_11_^–^	471.09328	471.09219	2.31	439 (50), **287** (100), 269 (20), 169 (20)	243 (15), 161 (20), **125** (100)	57 (100)	–	+	+	–	–
**64**	7.68	**(Epi)catechin 3-*O*-vanillate**	C_23_H_19_O_9_^–^	439.10346	439.10275	1.62	**289** (100), 287 (90), 271 (50), 269 (30), 167 (10)	**245** (100), 205 (30), 179 (15)	227 (30), 203 (100), 187 (30), 175 (10), 161 (20)	–	+	–	–	–
**65**	8.12	**(Epi)catechin 3-*O*-coumarate**	C_24_H_19_O_8_^−^	435.10854	435.10828	0.60	341 (15), **289** (100), 269 (25), 165 (20)	**245** (100), 205 (30), 179 (15)	227 (30), 203 (100), 187 (30), 175 (10), 161 (20)	–	+	–	–	–
	***Flavanols***													
**66**	6.09	**Myricetin 7-*O*-pentoside**	C_20_H_17_O_12_^–^	449.07255	449.07162	2.07	**317** (100)	299 (10), 289 (60), **273** (100), 255 (40)	255 (100), 211 (90)	–	–	–	+	+
**67**	6.17	**Myricetin *^a^***	C_15_H_9_O_8_^−^	317.03029	317.02942	2.74	299 (10), 273 (35), **207** (100), 163 (95)	**179** (100), 151 (15)	151 (100)	–	–	–	+	+
**68**	6.20	**Myricetin 3-*O*-hexoside**	C_21_H_19_O_13_^–^	479.08311	479.08176	2.82	317 (90), **316** (100)	287 (30), **271** (100), 179 (20), 151 (10)	271 (10), 243 (100), 227 (30), 215 (10), 199 (10)	+	–	–	+	+
**69**	6.21	**Quercetin 3-*O*-hexuronide-7-*O*-hexoside**	C_27_H_27_O_18_^–^	639.12029	639.11902	1.99	505 (10), **477** (100), 463 (30), 301 (50)	**301** (100)	273 (20), 257 (20), 179 (100), 151 (75)	+	–	–	–	+
**70**	6.23	**Quercetin 3-*O*-(6”-rhamnosyl)hexoside *^a^***	C_27_H_29_O_16_^–^	609.14611	609.14508	1.69	343 (5), **301** (100), 300 (30), 271 (10), 255 (5)	273 (25), 257 (20), **179** (100), 151 (75)	151 (100)	–	–	–	+	+
**71**	6.38	**Myricetin 3-*O*-hexuronide-7-*O*-hexoside**	C_27_H_27_O_19_^–^	655.11520	655.11359	2.46	521 (20), **493** (100), 479 (30), 317 (50)	**317** (100)	193 (10), 179 (100), 151 (30)	–	–	–	+	+
**72**	6.60	**Myricetin 7-*O*-hexuronide**	C_21_H_17_O_14_^–^	493.06238	493.06100	2.80	359 (10), **317** (100)	**179** (100), 151 (40)	151 (100)	–	–	–	+	+
**73**	6.67	**Quercetin 3-*O*-galactoside *^a^***	C_21_H_19_O_12_^–^	463.08820	463.08655	3.56	**301** (100), 300 (30)	273 (25), 257 (20), **179** (100), 151 (75)	151 (100)	+	+	+	+	+
**74**	6.81	**Kaempferol 7-*O*-(6”-rhamnosyl)hexoside**	C_27_H_29_O_15_^–^	593.15119	593.14905	3.61	**285** (100)	267 (40), **257** (100), 241 (30), 229 (40), 213 (30)	255 (10), 239 (30), 229 (100), 163 (40)	–	–	–	–	+
**75**	6.88	**Isorhamnetin 3-*O*-(6”-rhamnosyl)hexoside**	C_28_H_31_O_16_^–^	623.16176	623.15973	3.26	**315** (100), 300 (20), 271 (10), 255 (5)	**300** (100), 287 (5), 272 (5)	271 (100), 255 (50), 151 (5)	–	–	–	–	+
**76**	6.99	**Kaempferol 3-*O*-glucoside *^a^***	C_21_H_19_O_11_^–^	447.09329	447.09219	2.46	327 (20), 285 (80), **284** (100), 255 (10)	**255** (100), 227 (10)	227 (100), 211 (60)	+	–	–	+	+
**77**	6.99	**Syringetin 3-*O*-hexoside**	C_23_H_23_O_13_^–^	507.11441	507.11346	1.87	479 (10), 387 (20), 345 (80), 345 (60), **344** (100), 299 (15)	330 (90), **316** (100), 301 (90), 287 (10), 273 (70)	301 (100), 300 (20), 287 (10), 273 (60)	+	–	–	+	–
**78**	7.01	**Isorhamnetin 3-*O*-hexoside**	C_22_H_21_O_12_^–^	477.10385	477.10275	2.31	357 (20), 315 (50), **314** (100), 300 (5), 299 (5)	300 (30), **285** (100), 271 (75), 257 (10), 243 (25)	270 (100)	–	+	+	+	–
**79**	7.11	**Quercetin 3-*O*-hexuronide**	C_21_H_17_O_13_^–^	477.06692	477.06503	3.96	**301** (100)	273 (20), 257 (20), **179** (100), 151 (75)	151 (100)	+	+	+	+	+
**80**	7.24	**Quercetin 3-*O*-hexuronide methyl ether**	C_22_H_19_O_13_^–^	491.08311	491.08102	4.26	473 (10), 315 (50), **301** (100), 300 (60)	283 (15), 272 (20), 256 (10), **179** (100), 151 (75)	151 (100)	–	–	–	+	+
**81**	7.36	**Quercetin 3-*O*-(6”-malonyl)hexoside**	C_24_H_21_O_15_^–^	549.08859	549.08710	2.71	**505** (100)	463 (30), **301** (100), 300 (50)	273 (15), 257 (15), 179 (100), 151 (85)	–	–	–	–	+
**82**	7.44	**Kaempferol 7-*O*-hexuronide**	C_21_H_17_O_12_^–^	461.07200	461.07101	2.15	**285** (100)	267 (40), **257** (100), 241 (30), 229 (50), 213 (25)	255 (10), 239 (30), 229 (100), 163 (60)	+	–	–	+	+
**83**	7.73	**Kaempferol 3-*O*-hexuronide methyl ether**	C_22_H_19_O_12_^−^	475.08820	475.08667	3.22	327 (10), 301 (10), 285 (70), **284** (100), 255 (35)	**255** (100), 227 (10)	227 (100), 211 (60)	–	–	–	–	+
**84**	7.84	**Kaempferol 7-*O*-(6”-malonyl)hexoside**	C_24_H_21_O_14_^–^	533.09368	533.09204	3.08	**489** (100)	**285** (100)	267 (50), 257 (100), 241 (40), 229 (60), 213 (30)	–	–	–	–	+
**85**	8.60	**Quercetin *^a^***	C_15_H_9_O_7_^−^	301.03537	301.03391	4.85	283 (15), 271 (60), 257 (25), **179** (100), 151 (80)	**151** (100)	107 (100), 83 (10)	+	+	–	+	+
**86**	9.49	**Kaempferol *^a^***	C_15_H_9_O_6_^−^	285.04046	285.03958	3.09	**255** (100), 227 (10)	**211** (100), 195 (5), 167 (15)	211 (40), 137 (100)	+	–	–	–	+
	***Stilbenes***													
**87**	7.11	**Resveratrol hexoside**	C_20_H_21_O_8_^−^	389.12419	389.12230	4.86	**227** (100)	**185** (100), 183 (40), 159 (35), 157 (30), 143 (20)	−	+	+	–	–	+
**88**	8.01	**Resveratrol *^a^***	C_14_H_11_O_3_^−^	227.07137	227.07054	3.66	**185** (100), 159 (30), 143 (20)	157 (10), **143** (100), 117 (5)	115 (100)	+	+	–	+	+
**89**	8.50	**Resveratrol tetramer**	C_56_H_41_O_12_^−^	905.26035	905.25586	4.96	**811** (100), 717 (50), 451 (15), 359 (15)	**717** (100), 357 (10)	699 (50), 675 (100), 623 (30), 611 (60), 357 (80)	+	+	–	–	–
**90**	8.97	**Resveratrol dimer**	C_28_H_21_O_6_^−^	453.13436	453.13229	4.57	435 (10), 369 (10), 359 (30), **347** (100), 333 (40)	329 (10), 305 (20), **253** (100), 240 (30), 225 (10)	225 (100), 209 (10)	+	+	–	–	–
**91**	9.07	**Resveratrol trimer**	C_42_H_31_O_9_^−^	679.19736	679.19412	4.77	**585** (100), 491 (10)	**491** (100), 479 (20), 385 (10)	473 (40), 447 (30), 421 (20), 397 (25), 385 (100)	+	+	–	–	–

*^a^* Confirmed using standards, the other compounds were identified according high resolution mass spectrometry (HRMS) and MS^n^; *t*_R_—retention time; Δ ppm—mean mass accuracy errors; + stands for detected and − stands for not detected compound.

**Table 3:** High resolution mass spectrometry (MS) data and positive ion MS^2^, MS^3^, and MS^4^ fragmentation of anthocyanins identified in Vranac wine (W) and berry skin (E) extracts.

No	t*_R_*, min	Anthocyanins	Molecular Formula, M^+^ (*m/z*)	Calculated Mass, M^+^ (*m/z*)	Exact Mass, M^+^ (*m/z*)	Δ ppm	MS^2^ Fragments, (% Base Peak)	MS^3^ Fragments, (% Base Peak)	MS^4^ Fragments, (% Base Peak)	W	E
	***Peonidin Derivatives***										
**1’**	3.95	**Peonidin 3,5-di-*O*-hexoside**	C_28_H_33_O_16_^+^	625.17631	625.17548	1.33	464 (20), **463** (100), 302 (10), 301 (60), 265 (5)	**301** (100)	286 (100), 258 (5)	−	+
**2’**	5.62	**Peonidin 3-*O*-hexoside isomer**	C_22_H_23_O_11_^+^	463.12349	463.12288	1.32	302 (10), **301** (100)	287 (10), **286** (100)	268 (20), 258 (100), 230 (25), 202 (5)	+	+
**3’**	5.15	**Peonidin 3-*O*-glucoside*^a^***	C_22_H_23_O_11_^+^	463.12349	463.12231	2.55	302 (10), **301** (100)	287 (10), **286** (100)	268 (20), 258 (100), 230 (25), 202 (5)	+	+
**4’**	5.70	**Peonidin 3-*O*-hexoside-pyruvate**	C_25_H_25_O_13_^+^	531.11332	531.11255	1.45	370 (15), **369** (100)	**354** (100), 326 (10)	336 (25), 326 (100), 298 (30), 253 (30)	+	+
**5’**	6.08	**Peonidin 3-*O*-(6”-acetyl)hexoside**	C_24_H_25_O_12_^+^	505.13405	505.13358	0.93	302 (10), **301** (100)	287 (10), **286** (100)	268 (20), 258 (100), 230 (25), 202 (5)	+	+
**6’**	6.48	**Peonidin 3-*O*-(6”-caffeloyl)hexoside**	C_31_H_29_O_14_^+^	625.15518	625.15369	2.38	317 (10), 302 (15), **301** (100), 286 (10)	287 (5), **286** (100)	268 (20), 258 (100), 257 (20), 230 (30), 202 (10)	−	+
**7’**	6.80	**Peonidin 3-*O*-(6”-*p*-coumaroyl)hexoside**	C_31_H_29_O_13_^+^	609.16027	609.15796	3.79	302 (10), **301** (100)	287 (10), **286** (100)	268 (20), 258 (100), 230 (25), 202 (5)	+	+
	***Delphinidin Derivatives***										
**8’**	4.50	**Delphinidin 3-*O*-glucoside*^a^***	C_21_H_21_O_12_^+^	465.10275	465.10123	3.27	304 (15), **303** (100)	285 (10), 275 (10), **257** (100), 247 (15), 229 (30)	239 (5), 229 (100), 213 (25), 201 (25), 173 (20)	+	+
**9’**	4.85	**Delphinidin 3-*O*-hexoside-pyruvate**	C_24_H_21_O_14_^+^	533.09258	533.09113	2.72	372 (10), **371** (100)	371 (30), 353 (10), 343 (10), **325** (100), 315 (10)	297 (100), 281 (80)	+	+
**10’**	5.45	**Delphinidin 3-*O*-(6”-acetyl)hexoside**	C_23_H_23_O_13_^+^	507.11332	507.11270	1.22	304 (10), **303** (100)	303 (20), 285 (10), **257** (100), 247 (10), 229 (30)	229 (100), 213 (25), 201 (30), 173 (15)	+	+
**11’**	6.22	**Delphinidin 3-*O*-(6”-*p*-coumaroyl)hexoside**	C_30_H_27_O_14_^+^	611.13953	611.13904	0.80	304 (10), **303** (100)	303 (20), 285 (10), **257** (100), 247 (10), 229 (30)	229 (100), 213 (25), 201 (30), 173 (15)	+	+
**12’**	6.53	**Delphinidin 3-*O*-hexoside isomer**	C_21_H_21_O_12_^+^	465.10275	465.10202	1.57	304 (15), **303** (100)	285 (60), 275 (10), **257** (100), 247 (30), 229 (80)	239 (5), 229 (100), 213 (5), 201 (10), 173 (5)	+	+
**13’**	6.56	**Delphinidin 3-*O*-hexuronide**	C_21_H_19_O_13_^+^	479.08202	479.08124	1.63	304 (10), **303** (100)	285 (50), 274 (20), **257** (100), 247 (30), 229 (80)	229 (100), 201 (10)	+	+
	***Malvidin Derivatives***										
**14’**	4.62	**Malvidin 3,5-di-*O*-glucoside *^a^***	C_29_H_35_O_17_^+^	655.18688	655.18469	3.34	494 (15), **493** (100), 332 (10), 331 (70)	**331** (100)	316 (80), 315 (100), 299 (90), 298 (30), 287 (70)	+	+
**15’**	5.35	**Malvidin 3-*O*-glucoside *^a^***	C_23_H_25_O_12_^+^	493.13405	493.13300	2.13	332 (10), **331** (100)	316 (80), **315** (100), 299 (90), 298 (30), 287 (70)	313 (90), 299 (25), 287 (100), 285 (70), 257 (60)	+	+
**16’**	5.66	**Malvidin 3-*O*-hexoside-acetaldehyde(Vitisin B)**	C_25_H_25_O_12_^+^	517.13405	517.13336	1.33	356 (15), **355** (100)	**339** (100), 322 (40), 311 (15), 294 (45), 266 (20)	337 (50), 321 (35), 311 (100), 309 (95), 293 (10)	+	+
**17’**	5.69	**Malvidin 3-*O*-pentoside**	C_22_H_23_O_11_^+^	463.12349	463.12347	0.04	332 (5), **331** (100), 301 (10)	316 (90), **315** (100), 299 (85), 298 (30), 287 (60)	313 (90), 299 (25), 287 (100), 285 (70), 257 (60)	+	+
**18’**	5.77	**Malvidin 3-*O*-hexoside-pyruvate(Vitisin A)**	C_26_H_25_O_14_^+^	561.12388	561.12244	2.57	400 (10), **399** (100)	**383** (100), 367 (70), 355 (30), 338 (80), 310 (40)	381 (60), 365 (40), 355 (100), 337 (15), 325 (20)	+	+
**19’**	5.91	**Malvidin 3-*O*-hexoside-acetone**	C_26_H_27_O_12_^+^	531.14970	531.14911	1.11	370 (10), **369** (100), 325 (5)	**353** (100), 336 (20), 325 (10), 308 (25), 280 (15)	351 (60), 335 (30), 325 (100), 323 (80), 295 (25)	+	+
**20’**	6.03	**Malvidin 3-*O*-(6”-acetyl)hexoside-pyruvate**	C_28_H_27_O_15_^+^	603.13445	603.13416	0.48	400 (15), **399** (100)	**383** (100), 367 (80), 355 (40), 338 (80), 310 (40)	381 (60), 365 (40), 355 (100), 337 (20), 325 (20)	+	−
**21’**	6.13	**Malvidin 3-*O*-(6”-acetyl)hexoside**	C_25_H_27_O_13_^+^	535.14462	535.14331	2.45	332 (10), **331** (100)	316 (80), **315** (100), 299 (90), 298 (30), 287 (70)	313 (100), 299 (30), 287 (90), 285 (80), 257 (60)	+	+
**22’**	6.39	**Malvidin 3-*O*-(6”-caffeoyl)hexoside**	C_32_H_31_O_15_^+^	655.16575	655.16406	2.58	332 (10), **331** (100)	316 (80), **315** (100), 299 (90), 298 (30), 287 (70)	313 (90), 299 (25), 287 (100), 285 (70), 257 (60)	−	+
**23’**	6.70	**Malvidin 3-*O*-(6”-*p*-coumaroyl)hexoside-pyruvate**	C_35_H_31_O_16_^+^	707.16066	707.15979	1.23	400 (10), **399** (100)	**383** (100), 367 (70), 355 (30), 338 (80), 310 (40)	381 (60), 365 (40), 355 (100), 337 (15), 325 (20)	+	+
**24’**	6.76	**Malvidin 3-*O*-hexoside-4-vinylcatechol (Pinotin A)**	C_31_H_29_O_14_^+^	625.15518	625.15440	1.25	464 (15), **463** (100)	448 (50), **447** (100), 419 (10), 402 (20), 374 (20)	447 (50), 445 (65), 429 (20), 419 (100), 417 (95)	+	−
**25’**	6.84	**Malvidin 3-*O*-(6”-*p*-coumaroyl)hexoside**	C_32_H_31_O_14_^+^	639.17083	639.16996	1.36	332 (10), **331** (100)	316 (80), **315** (100), 299 (90), 298 (30), 287 (70)	313 (90), 299 (25), 287 (100), 285 (70), 257 (60)	+	+
**26’**	6.89	**Malvidin 3-*O*-(6”-feruloyl)hexoside**	C_33_H_33_O_15_^+^	669.18140	669.17857	4.23	332 (10), **331** (100)	316 (80), **315** (100), 299 (90), 298 (30), 287 (70)	313 (90), 299 (25), 287 (100), 285 (70), 257 (60)	−	+
**27’**	6.99	**Malvidin 3-*O*-hexoside-4-vinylphenol**	C_31_H_29_O_13_^+^	609.16027	609.16022	0.08	448 (25), **447** (100)	432 (50), **431** (100), 414 (15), 386 (20), 358 (20)	431 (50), 429 (65), 413 (20), 403 (100), 401 (95)	+	−
**28’**	7.19	**Malvidin 3-*O*-hexoside-pyranone**	C_25_H_25_O_13_^+^	533.12897	533.12677	4.13	372 (10), **371** (100)	356 (15), **343** (100), 339 (10), 311 (30), 283 (20)	311 (100), 283 (60), 265 (5), 255 (15)	+	−
**29’**	7.30	**Malvidin 3-*O*-(6”-acetyl)hexoside-4-vinylphenol**	C_33_H_31_O_14_^+^	651.17083	651.17035	0.74	448 (25), **447** (100)	432 (50), **431** (100), 414 (15), 403 (10), 386 (20)	431 (50), 429 (65), 413 (20), 403 (100), 401 (95)	+	−
**30’**	7.40	**Malvidin-pyruvate**	C_20_H_15_O_9_^+^	399.07106	399.07031	1.88	382 (20), **381** (100)	366 (10), **353** (100), 337 (10), 325 (90), 297 (60)	338 (5), 325 (100), 309 (10), 297 (25)	+	−
**31’**	7.55	**Malvidin 3-*O*-(6”-*p*-coumaroyl)hexoside-4-vinylphenol**	C_40_H_35_O_15_^+^	755.19705	755.19592	1.50	448 (25), **447** (100)	432 (50), **431** (100), 414 (15), 403 (10), 386 (20)	431 (50), 429 (65), 413 (20), 403 (100), 401 (95)	+	−
**32’**	7.30	**Malvidin 3-*O*-(6”-*p*-coumaroyl)hexoside-4-vinylcatechol**	C_40_H_35_O_16_^+^	771.19196	771.19055	1.83	464 (15), **463** (100)	448 (50), **447** (100), 430 (15), 419 (10), 402 (20)	447 (50), 445 (65), 429 (20), 419 (100), 417 (95)	+	−
	***Cyanidin Derivatives***										
**33’**	4.78	**Cyanidin 3-*O*-glucoside *^a^***	C_21_H_21_O_11_^+^	449.10784	449.10684	2.23	288 (10), **287** (100)	269 (25), 259 (40), 241 (50), 231 (70), **213** (100)	195 (10), 185 (100), 167 (10), 157 (20), 141 (20)	+	+
**34’**	5.04	**Cyanidin 3-*O*-arabinoside *^a^***	C_20_H_19_O_10_^+^	419.09727	419.09613	2.72	288 (10), **287** (100)	269 (25), 259 (40), 241 (50), 231 (70), **213** (100)	195 (10), 185 (100), 167 (10), 157 (20), 141 (20)	+	+
**35’**	6.43	**Cyanidin 3-*O*-(6”-*p*-coumaroyl)hexoside**	C_30_H_27_O_13_^+^	595.14462	595.14374	1.48	288 (10), **287** (100)	269 (25), 259 (40), 241 (50), 231 (70), **213** (100)	195 (10), 185 (100), 167 (10), 157 (20), 141 (20)	−	+
	***Petunidin Derivatives***										
**36’**	4.89	**Petunidin 3-*O*-hexoside**	C_22_H_23_O_12_^+^	479.11840	479.11655	3.86	318 (10), **317** (100)	**302** (100), 274 (5)	274 (100), 246 (10), 228 (10), 218 (10)	+	+
**37’**	5.79	**Petunidin 3-*O*-(6”-acetyl)hexoside**	C_24_H_25_O_13_^+^	521.12897	521.12872	0.48	318 (10), **317** (100)	**302** (100), 285 (30), 274 (10)	285 (10), 274 (100), 246 (15), 153 (20)	+	+
	***Pelargonidin Derivatives***										
**38’**	5.17	**Pelargonidin 3-*O*-(6”-rhamnosyl)glucoside *^a^***	C_27_H_31_O_14_^+^	579.17083	579.16989	1.62	434 (5), 433 (15), 272 (10), **271** (100)	**271** (100), 253 (40), 225 (60), 215 (70), 197 (80)	197 (5), 169 (100), 153 (5), 141 (30)	+	+

*^a^* Confirmed using standards, the other compounds were identified according to high resolution mass spectrometry (HRMS) and MS^n^; *t*_R_—retention time; Δ ppm—mean mass accuracy errors; + stands for detected and − stands for not detected compound.

## Supplementary Materials

The following are available online at https://www.mdpi.com/2304-8158/9/2/138/s1, Figure S1: sketch of all three vineyards.
