# Supplementary figures and images for: Phenolic Profiles of Leaves, Grapes and Wine of Grapevine Variety Vranac (Vitis vinifera L.) from Montenegro
Source: Foods. 2020 Jan 28;9(2):138. doi: 10.3390/foods9020138 (PMC7073729; doi:10.3390/foods9020138)

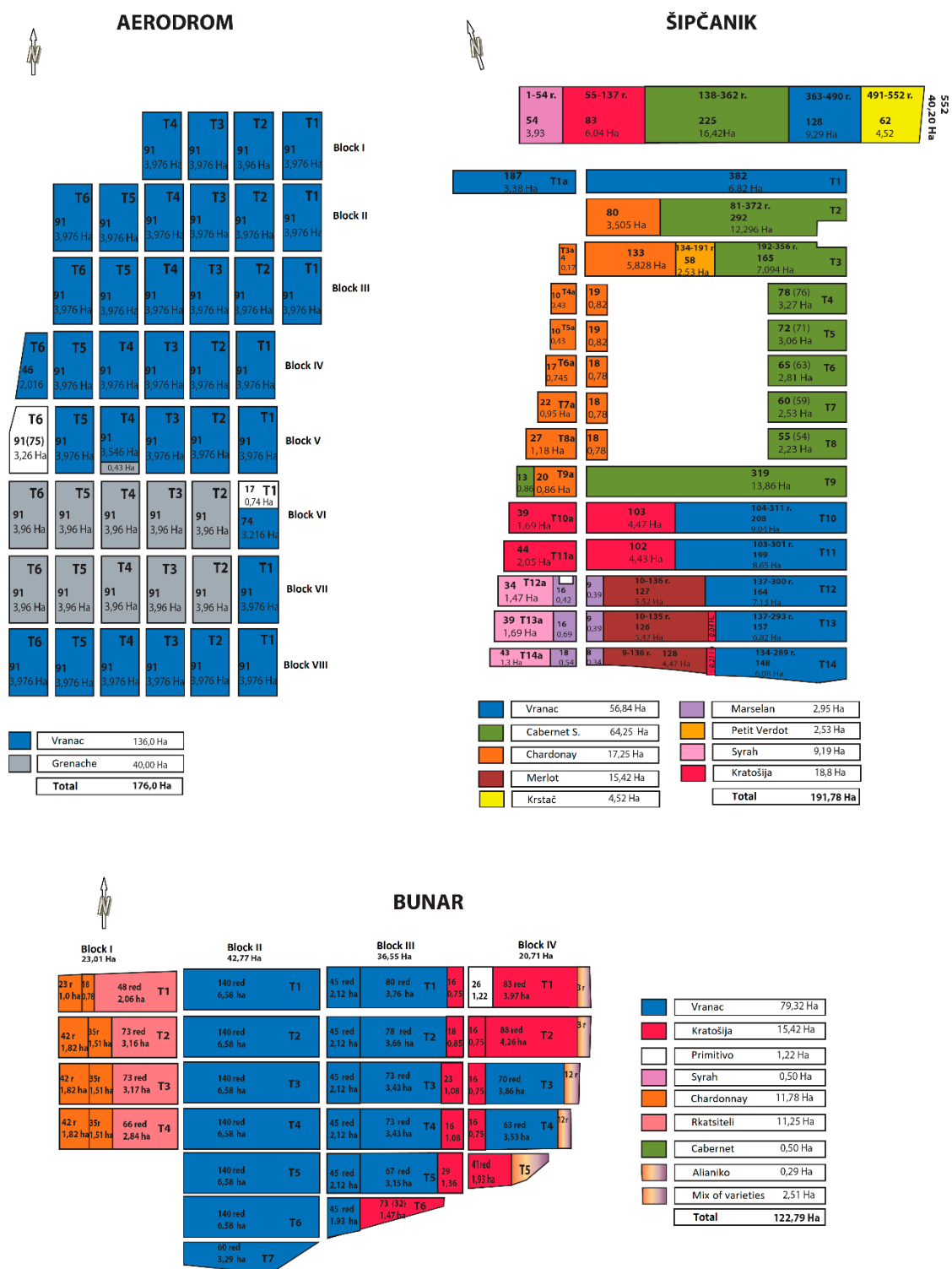

Figure S1. sketch of all three vineyards.

Supplement: Supplementary file 1 [file foods-09-00138-s001.pdf]
